# Gene variants for the WNT pathway are associated with severity in periodontal disease

**DOI:** 10.1007/s00784-023-05436-x

**Published:** 2024-02-06

**Authors:** María-Victoria Ospina-Ch, Mónica Acevedo-Godoy, Sandra J. Perdomo, Lorena Chila-Moreno, Gloria I. Lafaurie, Consuelo Romero-Sánchez

**Affiliations:** 1 School of Dentistry, Periodontics and Oral Medicine Program, Universidad El Bosque, Av. Cra. 9 #131A–02, Bogotá, Colombia; 2 Rheumatology and Immunology Department Hospital Militar Central/School of Medicine, Clinical Immunology Group, Universidad Militar Nueva Granada, Transversal 3ª # 49-00, Bogotá, Colombia; 3 Universidad El Bosque, Facultad de Ciencias, Maestría de Ciencias Básicas Biomédicas, Av. Cra. 9 #131A–02, Bogotá, Colombia; 4 School of Dentistry, Cellular and Molecular Immunology Group/ INMUBO, Universidad El Bosque, Av. Cra 9 No. 131 A–02, Bogotá, Colombia; 5 Universidad El Bosque, School of Dentistry, Unit of Oral Basic Investigation, UIBO Av. Cra. 9 #131A–02, Bogotá, Colombia

**Keywords:** Polymorphism, Single nucleotide polymorphisms (SNPs), Periodontal disease, Wnt

## Abstract

**Objective:**

Studies of Wnt variants-related to bone resorption in periodontitis are limited. The aim of this study was to establish the genotype and allele frequency of gene variants associated with the Wnt pathway in systemically healthy individuals with and without periodontitis (PD).

**Materials and methods:**

One hundred fifty-seven systemically healthy individuals were evaluated, 90 with PD and 67 without PD. Periodontal clinical indexes, serological and clinical indices of inflammation, and the following variants associated with the Wnt pathway: DKK, SOST, LRP5, and KREMEN were analyzed by high resolution melting and confirmed by Sanger sequencing.

**Results:**

In the PD-free group, 67.2% of the individuals presented the variant for DKKrs1896367 (p = 0.008) and 82.6% had the variant for KREMEN rs132274 (p = 0.016). The heterozygous variant for the DKK rs1896367 polymorphism was associated with the absence of PD and lower severity OR: 0.33 (CI95% 0.15–0.70) and OR: 0.24 (CI95% 0.11–0.53), respectively. Similarly, KREMEN rs132274 was the homozygous variant associated with the absence of PD (OR: 0.33 (CI95% 0.13–0.88)). On the contrary, 85.6% of individuals with PD presented a variant for DKK rs1896368 (p = 0.042), all suffering severe forms of periodontitis.

**Conclusion:**

The presence of DKKrs1896367 and KREMENrs132274 variants in individuals without PD suggests that these single nucleotide polymorphisms could be protective factors for bone loss in PD. A very interesting finding is that the DKKrs1896368 variant was found in a high percentage of severe cases, suggesting that the presence of this variant may be related to the severe bone loss observed in PD.

**Supplementary Information:**

The online version contains supplementary material available at 10.1007/s00784-023-05436-x.

## Introduction

Periodontitis (PD) is a chronic multifactorial disease associated with the dysbiosis of subgingival biofilm and causes inflammation within the tissues that support the teeth, eventually resulting in bone loss [1]. PD induces an inflammatory response in the gingival tissue and periodontium that results in the production of cytokines by inflammatory cells and the activation of signaling pathways promoting bone tissue resorption, leading to clinical insertion loss and bone loss [2].

Several signaling pathways are understood to play a role in bone resorption in PD, among which are the pathways related to the RANK/RANKL/OPG axis [3, 4] and the Wingless* e Int* Wnt/beta-catenin pathway, which critically modulate bone homeostasis through regulation of the RANK/RANKL/OPG axis [3, 5–7]. This pathway is the focus of research in various pathologies such as bone cancer, rheumatoid arthritis (RA), and osteoporosis, as well as diseases that affect bone density [7–9]. Literature has identified that an imbalance in the canonical Wnt pathway caused by an alteration in the genetic sequence of its components affects bone homeostasis [10].

Activating proteins known as Wnt ligands bind to their receptors, which activate the assembly of an intracellular pathway responsible for activating an osteoblastic profile. Antagonist molecules of this pathway include sclerostin (SOST), KREMEN, and dickkopf (DKK), which bind to their receptors and inhibit the cytoplasmic accumulation of beta-catenin and as a result osteoblastogenesis [11]. It has been proposed that alterations in the genetic sequence of this pathway, such as a mutation or a genetic variant, can be genetic risk factors for pathological entities characterized by bone loss. Therefore, the genetic polymorphisms related to the production of these proteins could become decisive in bone modulation in PD [9]. However, the literature that relates this pathway to PD is very scarce. Local and circulating levels of the SOST and DKK proteins have been found in systemically healthy subjects with chronic periodontitis, suggesting these proteins may be involved in periodontal diseases [4]. Furthermore, single nucleotide polymorphisms (SNPs) related to components of this pathway are described in inflammatory systemic diseases such as Rheumatoid Arthritis (RA). Rooy et al. [12] have suggested that different variants are related to greater bone erosion.

Given the limited information available on the role of this signaling pathway in PD, this study aimed to establish the genotype and allele frequency of gene variants associated with the Wnt pathway in systemically healthy Colombian individuals with and without PD.

## Materials and methods

This was a cross-sectional study carried out in the city of Bogotá, Colombia, where 157 systemically healthy individuals from Bogotá-Colombia aged between 30 to 65 were classified into two groups: 90 individuals were diagnosed with PD and 67 individuals without PD, according to the Centers of Disease Control/CDC and American Academy of Periodontology/AAP criteria [13, 14], The inclusion criteria of healthy individuals were adults from the general population, men or women over 18 and under 65 years of age.

The screening criteria, inflammatory markers, periodontal clinical, immunological and microbiological parameters, bone marker polymorphisms in the Wnt pathway, automated DNA sequencing and statistical analysis are described in Supplementary Annex 1.

## Results

### Demographic and clinical characteristics of the population

Of the 157 individuals evaluated periodontally, based on the CDC classification 57.3% were diagnosed with PD, 8.9% of whom showed mild severity, 38.9% moderate, and 9.6% were severe [14, 15] The individuals had an average age of 41.1 ± 13.08, and 73.9% (116) were women, and 26.1% (41) were men. Although this population had apparently healthy individuals, 34.3% had at least one comorbidity, the most frequent ones being arterial hypertension (18.5%), osteoarthritis (16.6%), and hypothyroidism (12.9%). The median body mass index (BMI) was 24 kg/m^2^ (range: 22–25.9), with 65.6% of the individuals falling in the normal range, 28.7% were categorized as overweight, and 5.7% as obese. Among the group, only 10.19% were smokers (Table 2 Supp).

### Inflammatory biomarkers

Inflammatory serological biomarkers showed normal ranges for ESR at 83.44% with a median of 6 mm/cm^3^ (Range: 2–13). Most of the subjects in this study had C-reactive protein levels within normal ranges (69.43%), and 24.20% had levels greater than 3 mg/L. The evaluation of arthritis markers showed negative levels for RF in 92.36% of individuals and positive (low) levels in 7.01%, with a mean for the total population of 11.80 ± 14.3 U (Table 2 Supp). Clinical analysis did not reveal any painful and swollen joints in the population (Data not shown).

### Periodontal condition

Individuals with PD had a poorer periodontal index than the population with healthy gingivitis (P < 0.0001), but there were no differences in the number of teeth. However, PD individuals had more frequent occurrences of *P. gingivalis* in the subgingival biofilm (Table 1).

**Table 1:** Periodontal indexes in health-gingivitis and periodontitis group

Periodontal indices	Health-gingivitis n = 67	Periodontitis n = 90	*p* value
*P. gingivalis* F (%) Presence Absence	23 (34.3) 44 (65.7)	48 (53.3) 52(46.7)	0.0150*
Number of Teeth Median (IQR)	27 (21–28)	25 (22–28)	0.2500
PI % Median (IQR)	36 (21–58)	54 (33–78)	< 0.0001**
GI% Median (IQR)	21 (7–37)	29 (16–57)	< 0.0001**
BOP % Median (IQR)	27 (5–41)	42 (23–58)	< 0.0001**
PD mm Median (IQR)	1.98 (1.7–2.16)	2.18 (1.96–2.53)	< 0.0001**
CAL mm Median (IQR)	0.4 (0.14–.1)	1.39 (0.85–2.15)	< 0.0001**

*PI* = Plaque Index; *GI* = Gingival Index; *BOP* = Bleeding on probing; *PD* = Pocket depth; *CAL* = Clinical attachment level. % = expressed in percentages; mm = expressed in millimeter. *IQR* = interguartile range; percentille 25 – 75, ** *p* < 0.0001; * *p* < 0.05

### Genotype frequencies

The distributions of the six SNPs evaluated in the study population were consistent with the Hardy–Weinberg equilibrium (p > 0.05), suggesting that these groups belonged to the same Mendelian population. The frequencies of the DKK genotypes rs1896367, rs189636855, and rs1528873 were 41%, 80.25% and 48.41%, respectively. 72.61% of the individuals displayed the KREMEN SNP (rs132274) and 69.43% had the SOST SNP (rs6503475). As for LRP5, 21% presented the SNP, 5% being homozygous, and 15.9% were heterozygous. The analysis of gene polymorphisms showed combinations of SNPs in the population without establishing any significant associations. In this regard, 30 individuals (19.1%) had the three (3) SNPs for the DKK gene. Similarly, 21 individuals (13.3%) had the combination of the six SNPs evaluated, and two (2) individuals (1.27%) had the combination of DKK rs1896367 and DKK rs1896368. Interestingly, the combination of the three DKK SNPs and the KREMEN variant was observed in 9 individuals (5.7%); whereas the presence of SNPs **for** DKK, KREMEN and SOST was observed in 21 individuals (13.3%).

### Comparison of variant genotype frequencies between the PD group and the PD-free group

**Fig. 1:**
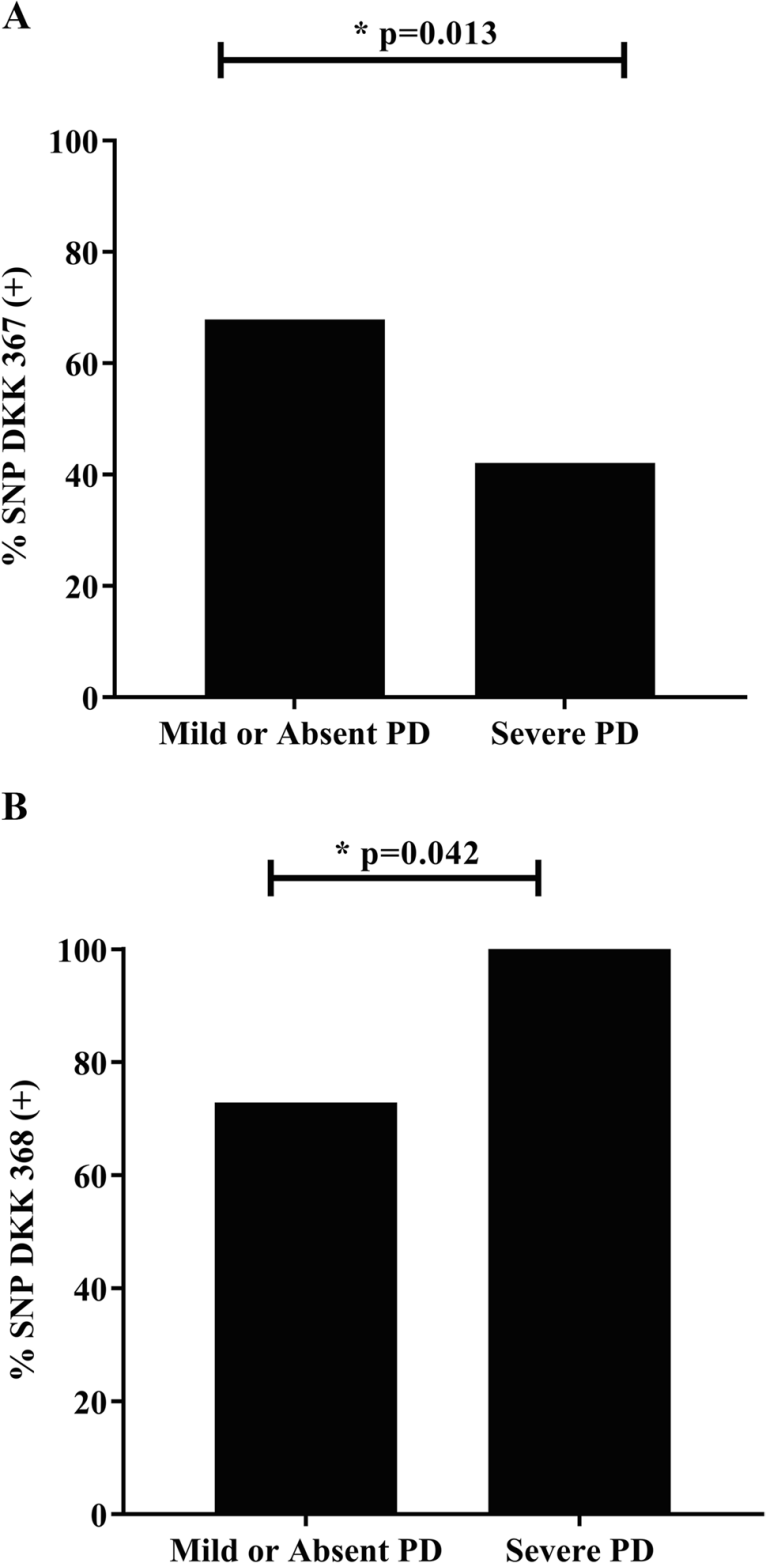
**a** Frequency analysis of SNP DKK 1896367 in individuals with PD according to severity levels. Data analyzed in GraphPad Prism V9.0. **b** Frequency analysis of SNP DKK rs1896367 in individuals with PD according to the new AAP classification

**Fig. 2:**
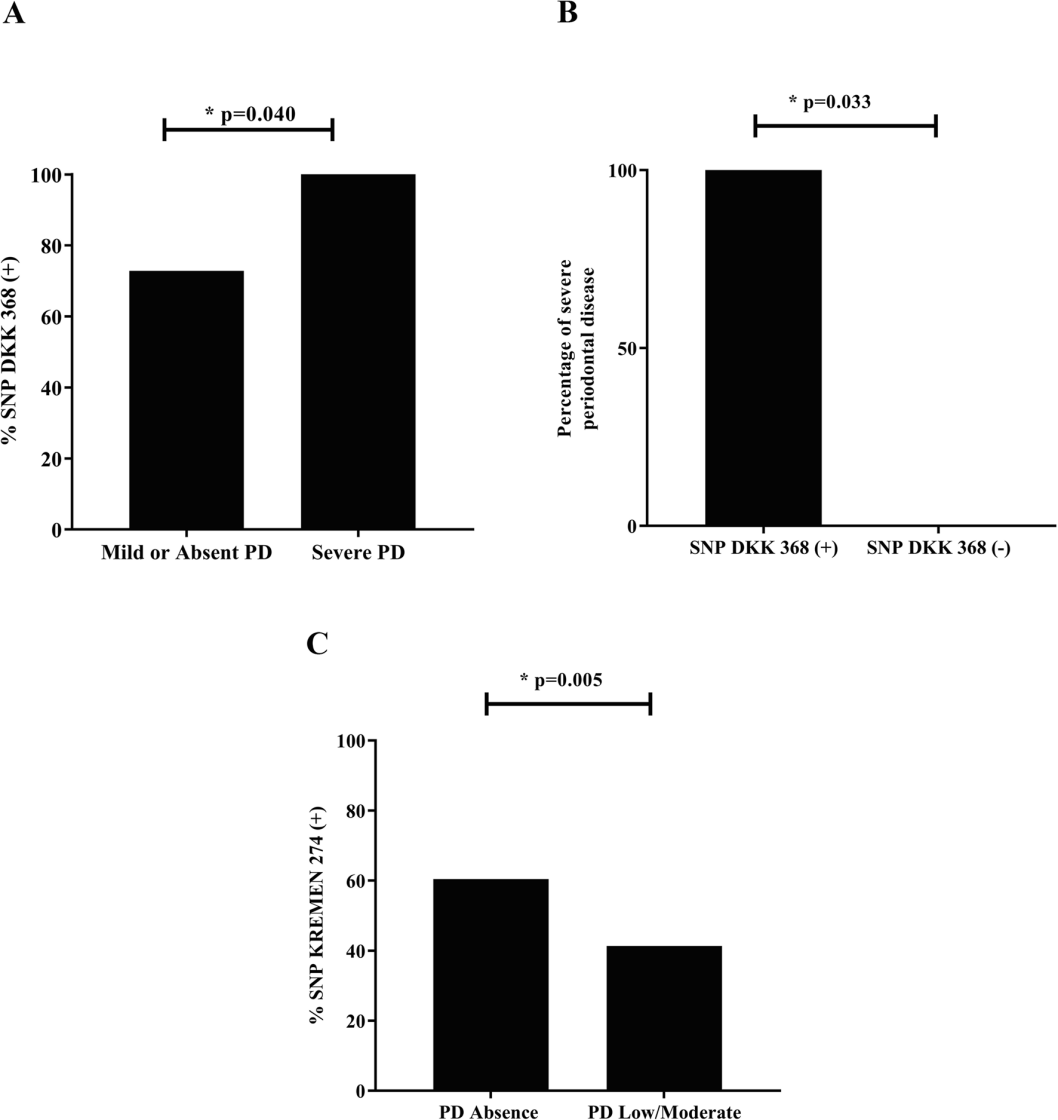
**a** Frequency analysis of SNP DKK 1896368 in individuals with PD according to severity level. **b** Percentage of individuals diagnosed with severe PD with a variant for DKK 1896368, % of severity level according to CDC. **c** Frequency analysis of SNP KREMEN rs132274 in individuals with PD

Positive associations were found between SNP DKK rs1896367 and KREMEN rs132274 and periodontal health (p = 0.013 and p = 0.005, respectively) (Figs. 1a and 2c). An association was observed between the variants of the DKK rs1896367 polymorphisms and KREMEN rs132274 and the diagnosis of PD in individuals older than 45 years where *P. gingivalis* was present (Table 2). Significant associations were also observed between the presence of the heterozygous state for the variant DKK rs1896367 (OR, 0.33 95% CI, 0.15–0.70) and KREMEN rs132274 in the homozygous state (OR: 95% CI 0.33 (0.13–0.88) with the diagnosis of PD (Table 3). Furthermore, an analysis of the presence of SNP DKK rs1896367 versus the severity of PD found that it prevailed in individuals with low degrees of or no severity (63.2%) (Fig. 1a), which is associated with the heterozygous state OR. 0.24 CI 95% (0.11–0.53) (Table 3 Supp).

**Table 2:** Genotype association of variants DKK rs1896367 and KREMEN rs132274 with the presence of periodontal disease

Dependent Variable	Reference	Unadjusted OR CI 95%	Adjusted OR CI 95%
Periodontitis diagnosis			
Model 1			
DKK rs1896367 positive	Negative	0.34 (0.36–0.80)	0.48 (0.16–1.46)
Age > 45 years	< 45 years	3.52 (1.71–7.2)	4.95 (1.68–14.5)
*P. gingivalis*	Negative	2.70 (1.31–5.5)	3.14 (1.08–9.11)
Model 2			
KREMEN rs132274 positive	Negative	0.38 (0.16–0.87)	1.22 (0.69–2.17)
Age > 45 years	< 45 years	3.18 (1.56–3.47)	1.71 (0.42–6.93)
*P. gingivalis*	Negative	2.59 (1.27–5.26)	3.87 (0.71–20.9)

Model 1: Adjusted by age and *P. gingivalis*. Adjusted by age, *P. gingivalis*, smoking history, and BMI Likelihood ratio = 0.66. BIC unadjusted model = 193.68, BIC adjusted model = 196.85. The unadjusted model must be reported. Model 2: Adjusted by age and *P. gingivalis*. Likelihood ratio = 0.55. BIC unadjusted model = 197.12, BIC adjusted model = 199.93. The unadjusted model must be reported

**Table 3:** Genotype association of heterozygous DKK rs1896367 and homozygous KREMEN rs132274 with the presence of periodontal disease

Dependent variable	Reference	Unadjusted OR CI 95%	Adjusted OR CI 95%
Periodontitis diagnosis			
Model 1 Independent variable			
DKK rs1896367 AA DKK rs1896367 AG	Negative Negative	0.83 (0.24–2.88) 0.33 (0.15–0.70)	1.11 (0.29–4.18) 0.62 (0.19–2.01)
Age > 45 years	< 45 years	3.48 (1.69–7.18)	6.47 (1.93–21.6)
*P. gingivalis*	Negative	2.68 (1.30–5.52)	3.86 (1.18–12.6)
Model 2 Independent variable			
KREMEN rs132274 TT KREMEN rs132274 CT	Negative Negative	0.33 (0.13–0.88) 0.41 (0.17–1.00)	0.35 (0.11–1.07) 0.65 (0.17–2.47)
Age > 45 years	< 45 years	3.06 (1.48–6.32)	3.40 (0.95–12.1)
*P. gingivalis*	Negative	2.62 (1.29–5.35)	5.12 (1.29–20.3)

Model 1: Adjusted by age, *P. gingivalis*, smoking history, and BMI. Likelihood ratio = 0.33. BIC unadjusted model = 206.16, BIC adjusted model = 214.09. The unadjusted model must be reported. Model 2: Adjusted by age and *P. gingivalis*, smoking history, and BMI. Likelihood ratio = 0.24. BIC unadjusted model = 214.12, BIC adjusted model = 221.39. The unadjusted model must be reported

PD have significant association between DKK rs1896368 and the disease was also observed (p = 0.042) (Fig. 1b). Furthermore, 46.6% being heterozygous and 38.9% homozygous. No differences were found for the SOST 475 and LRP5 228 variants. Significant associations were observed between the SNP DKK rs1896368 and individuals with severe PD (100%, p = 0.040) and moderate PD (82.2%, p 0.033) (Fig. 2a, b). The KREMEN variant showed a significant association in individuals with less severity (p = 0.005) (Fig. 2c).

The DKK variant rs1896368 predominated in individuals diagnosed with PD (n = 49, 73.1%); however, interestingly, it predominated in moderate and severe PD (p = 0.034). As for the KREMEN gene variant, 55 individuals with this variant (82.1%) were not diagnosed with PD; The presence of SNP rs1896368 was associated with the periodontal clinical indicator CAL with a median ≥ 3 mm of 16.67 (4.17–32.62) and for ≥ 4 mm of 3.77 (0.56–14. 04) being higher in individuals who did not present the SNP (p = 0.003) (Table 4 Supp).

### Allele frequencies for variants of the Wnt pathway

The distribution of genotype and allele frequency in the evaluated SNPs is summarized in Table 1 Supp. The DKK rs1896367 T allele indicated a MAF of 0.32, the DKK rs1896368 C allele indicated a MAF of 0.57, and the DKK rs1528873 C allele indicated a MAF of 0.25. For the SNP KREMEN rs132274, a MAF of 0.55 was recorded for the T variant. Similarly, for SOST rs6503475, the frequency of the G allele was 0.44 (43.63%), and for LRP5 rs3736228, the MAF was 0.21 (21.01%) for the T allele.

### Allele frequency analysis of variants and their association with periodontal diagnostic parameters

Individuals carrying the minor T allele of DKK rs1896367 (49.5%) presented PD (p = 0.054), unlike individuals with the ancestral C allele. No association was observed between the presence of *P. gingivalis* and IgG1 antibody titers against the periodontopathogen. Individuals with IgG2 against *P. gingivalis* carried the rs1896367 variant at a frequency of 71.3% (p = 0.037).

Lastly, analysis of the allele frequencies of the SNP DKK rs1896367 against the periodontal clinical variables, only revealed association with sites that demonstrated CAL ≥ 3 mm in individuals who displayed the ancestral G alleles with a median of 16.07 (3.57–32.62), as opposed to a median of 8.89 (0.60–26.85) in individuals who presented the variant A allele, with a significant p value (p = 0.036).

Similarly, an analysis of allele frequencies of the SNP KREMEN rs132274 found that there were 81 (50%) T alleles in the group of individuals diagnosed with PD, compared to 99 (65.1%) ancestral C alleles (p = 0.007). Regarding the analysis of the severity of PD, 72 (47.4%) ancestral C alleles were found within the group of individuals who presented moderate PD as opposed to 50 (39.9%) T alleles, leading to the idea that the ancestral allele is related to the presence of moderate PD (p = 0.007).

The DKK rs1896368 minor G allele was associated with the presence of a PD diagnosis (MAF 0.62); however, no association was found with periodontal clinical parameters such as the presence of *P gingivalis* or antibody titers against the bacteria. Regarding PD severity, it was observed that individuals with moderate and severe PD carried the minor G allele (MAF 0.12), which suggests that the presence of the G allele is related to the presence and severity of PD (p = 0.027).

The other analysis that yielded significant results compared to periodontal clinical variables was that of the SNP DKK rs1896368 allele frequencies and sites with CAL ≥ 4 mm in individuals with the ancestral A allele (p = 0.004).

Analysis of the allele frequencies for the DKK rs1528873, SOST rs6503475, and LRP rs3736228 variants, revealed no significant results.

## Discussion

PD is an infectious condition in the structures surrounding the tooth, which results from complex interactions between plaque microorganisms and the host’s immune system. This disease has been associated with the expression of mediators related to soft tissue destruction and bone loss, suggesting a dynamic process between microbial aggression and inflammatory responses [1, 3, 16, 17].

This study evaluated the possible involvement of SNPs of the DKK-1, SOST, KREMEN, and LRP5 genes in the Wnt/β-catenin signaling pathway in systemically healthy individuals—90 with PD and 67 control group individuals without PD. After binding to FZD and/or LRP5/6 receptors, WNT proteins activate the canonical Wnt/β-catenin signaling pathway, which in turn activates gene transcription [18] involved in adult bone tissue homeostasis and osteoblastogenesis [19]. Aberrant regulation of the signaling pathway owing to host genetic polymorphisms has been reported to influence resorption and/or remodeling of bone tissue and has been implicated in pathologies such as imperfect osteogenesis, RA, and osteoporosis [19, 20]. However, there is limited information on the role of such polymorphisms in PD.

The global genotype frequencies of these six polymorphisms were congruent with those reported for the Colombian population; the DKK rs1896368 C allele was the most frequent one (0.580), as previously reported in the Antioquia population in Colombia (0.510). Additionally, the allele frequencies for the DKK rs1896367 C and T alleles (0.670 and 0.320 respectively) were like those that have been previously reported in the Colombian population (0.610 and 0.380 respectively) [21]*,* suggesting little variability in the distribution of DKK alleles within the population sample. On the other hand, the allelic frequencies of the variant we identified were consistent with the findings on the Colombian population within the study of 1,000 genomes, for SOST rs6503475 G allele (0.430 and 0.450 respectively), as well as the T allele (0.210 and 0.154 respectively) and C allele (0.875 and 0.789 respectively) of LRP5 rs3736228 [21].

Combinations of genetic variants are typically considered as general risk factors for polygenic disorders, while the accumulation of combinations of variants in an individual’s genome are deemed personal risk factors [22]*,* suggesting that genetic factors may have a significant role in the etiology of certain diseases.

In this regard, the analysis of gene polymorphisms in the systemically healthy population revealed combinations of genotypes of the SNPs of DKK-1, DKK-1/KREMEN and DKK-1/Kremen/SOST that did not have a significant association with the diagnosis of PD, which suggests that these combinations may have little or no functional impact on the disease [23]*.* Thirty out of 157 individuals demonstrated three SNPs of DKK and KREMEN simultaneously, and 16 of those 30 have PD. Furthermore, another group of 32 patients (out of 157) simultaneously exhibited three variants of DKK + KREMEN + SOST; 17 of those 32 patients have PD.

However, one study’s main finding is that the SNPs DKK rs1896367 and KREMEN rs132274 occurred at a higher frequency (0.240 and 0.820 respectively) in systemically healthy individuals without PD, thus pointing to the potentially protective role of such variants. Interestingly, the absence of the KREMEN rs132274 variant was associated with a higher percentage of sites with CAL ≥ 2 mm. Additionally, the ancestral genotype DKK rs1896367-GG was the predominant one in this population, as reported in healthy control groups in the Acevedo 2018 study [24]*.* Severe PD was associated with the finding of the heterozygous genotype of the SNP DKK rs1896367 and the homozygous KREMEN rs132274, as reported by Cardona et al., in a Colombian population with RA and PD [25] and previously significantly associated with the progression of joint damage [12]. Furthermore, a predominance of the SNP DKK rs1896368 heterozygous genotype was observed in severe PD [25]*.* In this regard, SNP rs1896368 has been found to associated with increased levels of functional DKK-1, and clinically with bone resorption and joint damage in RA [25, 26]. Additionally, the elevated protein expression observed in gingival tissue and serum of patients with chronic periodontitis, suggests that DKK-1 is the main member of the dickkoff family that inhibits the Wnt pathway in PD [4]. Heredia-Palau et al. [27], who also studied the association between crevicular fluid levels of DKK and bone loss associated with the presence of PD in 24 patients with RA and a control group—found that both serum and crevicular fluid levels were associated with periodontal diagnosis and its severity [27] However, on analysis of factors associated with the progression of PD in this group of patients, the levels of DKK did not yield any significant data [28]. DKK-1 is a negative regulator of the Wnt-β-catenin signaling pathway; components of this pathway, including those encoded by DKK-1, SOST, LRP5, and KREMEN1, have well-known roles in osteoblastic differentiation [12].

In this study, the genetic variant of the DKK-1 rs1896368 gene was associated with a diagnosis of severe and moderate PD, suggesting that individuals with PD could carry risk alleles of genetic variants in *DKK-1*. Additionally, both the allele and the genotype *DKK-1* rs1896367 and the *KREMEN* SNP rs132274 presented a significant frequency in the group without PD, with a significant difference, which could suggest that the two variants may provide a measure of protection against PD for individuals who have them. Future prospective studies should be expanded and designed to gain better insights. These data illustrate the relevance of *DKK-1* in the progression of bone destruction associated with PD.

Moreover, no significant differences were observed between the presence of any SNP and *P. gingivalis* antibodies in healthy individuals, which is consistent with the findings of Cardona et al. on *DKK-1* SNPs in 63 patients in the early stages of RA and a healthy control group [25].

Our study is the first to show a relationship between the polymorphisms of the Wnt/B catenin pathway components in PD. However, more studies are needed to further investigate this association.

Furthermore, native genotypes for DKK rs1528873 have been defined, where the ancestral allele is reported as TT, and the variant or the presence of SNP corresponds to G. In the study carried out in a population in Antioquia, based on 188 reported alleles, the complementary allele frequencies reported for this SNP DKK rs1528873 found in the healthy population represents 0.5053 for the ancestral A allele, compared to our population, which had a higher allele frequency, with 234 ancestral alleles (0.74). This data highlights a difference between the frequencies previously found. Likewise, 49.47% (0.4947) of the Antioquia population had variant C, compared to 0.25 found in our study [21]. https://www.ncbi.nlm.nih.gov/variation/tools/1000genomes/*).* This represents an interesting difference compared to this study population, which included individuals from different regions of Colombia without being associated with any periodontal variable.

### Clinical relevance

Scientific Rationale for Study: Several signaling pathways are understood to play a role in bone resorption in PD. Literature has identified that an imbalance in the canonical Wnt pathway caused by an alteration in the genetic sequence of its components affects bone homeostasis.

Principal Findings: This study finds the presence of DKKrs1896367 and KREMENrs132274 variants in individuals without PD suggests that these single nucleotide polymorphisms could be protective factors for bone loss in PD. Otherwise, the DKKrs1896368 variant was found in a high percentage of severe cases, suggesting that the presence of this variant may be related to the severe bone loss observed in PD.

Practical Implications: The results are very interesting and potentially suggest the presence of some such variants could be related to the severity of bone loss that is characteristic of PD.

### Strengths and weaknesses

To our knowledge, this is the first study to analyze genotypes and allele variants of the genes related to the Wnt pathway in systemically healthy individuals in association with clinical, microbiological, and immunological variables of PD. The results are very interesting and potentially suggest the presence of some such variants could be related to the severity of bone loss that is characteristic of PD. Studies about protective factors may lead to studies on the Wnt pathway as related to this pathology and therapeutic interventions.

The main limitation of this study is the relatively small number of patients. This was in turn due to the difficulty in enrolling patients while following the strict inclusion criteria of the study.

### Supplementary Information

Below is the link to the electronic supplementary material.

Supplementary file1 (DOCX 62 KB)
