# Supplementary material for: Gene variants for the WNT pathway are associated with severity in periodontal disease
Source: Clin Oral Investig. 2024 Feb 6;28(2):135. doi: 10.1007/s00784-023-05436-x (PMC10847211; doi:10.1007/s00784-023-05436-x)
Supplement: Supplementary file 1 — Supplementary file1 (DOCX 62 KB) [file 784_2023_5436_MOESM1_ESM.docx]

**Supplementary material:**

**Supp information 1:**

This was a human observational study and complied the STROBE guidelines.

Individuals with ongoing infectious processes or with diagnoses of neoplasms, diabetes mellitus, autoimmune and systemic diseases, lactating women, those under antibiotic treatment for three months prior to sample collection, those who had orthodontic devices, and those who had undergone periodontal therapy in the past six months were excluded.

The rheumatologist collected demographic data, as well as clinical parameters associated with inflammation, from everyone, and evaluated painful or inflamed joints to rule out any compromised joints or bones. Likewise, laboratory tests associated with inflammation and joint involvement such as erythrocyte sedimentation rate (ESR), rheumatoid factor (RF), and anti-citrullinated peptide antibodies (anti-CCP) were performed. This study was approved by the Hospital Militar Central Research and Ethics Committee (2015-047) and was performed in accordance with the ethical standards as laid down in the 1964 Declaration of Helsinki and its later amendments or comparable ethical standards. All participants signed an informed consent form after receiving sufficient information and understanding the objectives of the study.

**Measurement of inflammatory markers: High Sensitivity C-Reactive Protein and erythrocyte sedimentation rate-ESR**

The C-reactive protein test was performed using chemiluminescence^§^ which produced a result in mg/L; results greater than 3 mg were considered as positive. The measurement of the ESR was performed by the photometric method, values greater than 20 mm were elevated^†.^

**Quantification of anti-citrullinated peptide autoantibodies and RF**

A sandwich-type ELISA system was used to quantitatively measure IgG/IgA against citrullinated peptides in serum^β^. The results were given in ELISA units/mL, where results greater than 20 IU were considered positive. RF was quantified by turbidimetry, where results greater than 20 U were considered positive^¥^.

**Periodontal clinical parameters and periodontal diagnosis**

The periodontal clinical parameters were evaluated by two (2) calibrated periodontists. The following parameters were evaluated: gingival Index [1], plaque index, pocket depth, clinical attachment loss (CAL), and bleeding on probing. Calibration for periodontal indices included probing depth, (with an inter-examiner correlation coefficient (IE-ICC) of 0.94–0.96); CAL ((IE-ICC) 0.92–0.96); bleeding on probing (BOP), ((IE-ICC) 0.88–0.90); plaque index (PI), ((IE-ICC) 0.94–0.98); and gingival index (GI), ((IE-ICC) 0.85–0.90), which were evaluated throughout the mouth including the selected sites. All measurements were performed by using a probe^¢^.

PD was defined as proximal CAL of at least 2 mm with a pocket depth of at least 4 mm, in two or more fully erupted teeth, The GI used was dichotomic. Depending on the severity of the disease, the individuals were grouped into one of three categories: mild, moderate, or severe periodontitis based on the Centers of Disease Control/CDC and American Academy of Periodontology/AAP criteria [2–4]. Individuals without PD were categorized as healthy or with gingivitis individuals.

**Evaluation of the presence of *Porphyromonas gingivalis* by** **Real-Time Polymerase Chain Reaction**

For everyone, the supragingival plaque was removed and absorbent paper stitches were inserted for 20 seconds at the six deepest periodontal sites. The primers and probes used for 16S homologs and rRNA sequences were selected with the aid of the MegAlign program, part of the Lasergene system^Ÿ^. PCR-based amplification was performed in a 25 µL total reaction mix. The samples were amplified using the following sequence: 95°C for 10 minutes, followed by 45 cycles at 95°C for 15 seconds, and at 60°C for one minute. Results for unknown plaque samples were projected onto a standard curve generated by using *P. gingivalis* (ATCC 33277), and then transformed into Log10 [5].

Evaluation of bone marker polymorphisms in the Wnt pathway**: DKK, SOST, LRP5, KREMEN**

Genomic DNA was extracted from EDTA anticoagulated whole blood using the DNA 2000 kit^α^ following the manufacturer’s directions. Briefly, the precipitate was washed with 70% ethanol, dissolved in 100 µL of reconstitution solution, and stored at −20°C until further use. Afterward, the bioinformatics tool BLAST https://blast.ncbi.nlm.nih.gov/Blast.cgi?PROGRAM=blastn&PAGE_TYPE=BlastSearch&LINK_LOC=blasthome) was used to evaluate the location of SNPs in genes and compare nucleotides [6]*.*

**Quantitative Polymerase Chain Reaction-high resolution melting Analysis (qPCR- HRM)**

The identification of variants was carried out through high resolution melting (HRM), using primers designed with the Beacon designer® program. PCR/HRM was performed using the Precision Melt Supermix kit^†^ by using samples with the Wild Type sequence as a control (Table 1 Supp). The PCR used a 20 µL reaction mix with 2 µM of each primer and 50 ng of DNA. Melting curve profiles were created and compared after amplification and analyzed with the Precision Melt Analysis ™ program^‡^. Product sizes were verified using 2% agarose gel electrophoresis.

**Automated DNA sequencing**

Random samples were selected for the verification process through Sanger sequences [7] and by runs on an Applied Biosystems 3500 Genetic Analyzer capable of reading sequences of approximately 1,000 base pairs^Š^.

**Statistical analysis**

The data distribution were evaluated to determine the non-parametric and parametric tests to be used. Chi-squared/fisher tests were used for categorical variable to establish the *p-*values. For the non-parametric tests, Kruskal–Wallis and Mann–Whitney U tests were used. Logistic regression was performed and confounding variables (age and presence of *P. gingivalis)* were adjusted to establish the association between bone variants and the appearance of periodontal disease. The “lrtest” likelihood method was implemented after each estimate to compare both the models (adjusted and not adjusted). The “fitstat” test, which compares both models while taking both using the Akaike information criterion and the BIC into account, was applied to confirm which model to choose. All analyses were performed by STATA for Windows with statistical significance set at p < 0.05

**LIST OF MATERIAL**

^§^ Immulite 1000, Siemmens® REF. LKCRP1 Erlangen Germany

^†^ (Ali- Fax) (Test 1 THL Ali-FAX®Polverara (PD)-Italy).

^β^ Quanta lite® CCP 3.1 IgG/IgA, INNOVA Diagnosis, San Diego, CA, USA and IMTEC–ITC 60015)

^¥^  RF TEST, ref. 1107105 Spinreact, Santa Coloma, Spain

^¢^ Hu-Friedy Mfg Co. Inc. Qulix™

^Ÿ^ DNAstar Inc., Madison, Wisconsin, USA

^α^ DNA200Corpogen Corporation ®, Bogotá Colombia

^Š^ Precision Melt Analysis ™ program Foster City, California, USA

**REFERENCES**

1. Löe H, Silness J (1963) Periodontal Disease in Pregnancy I. Prevalence and Severity. Acta Odontol Scand 21:533–551. https://doi.org/10.3109/00016356309011240

2. Kersley GD (1949) Dental Sepsis and Chronic Rheumatism. Proc R Soc Med 42:151–153. https://doi.org/10.1177/003591574904200315

3. Page RC, Eke PI (2007) Case Definitions for Use in Population-Based Surveillance of Periodontitis. J Periodontol 78:1387–1399. https://doi.org/10.1902/jop.2007.060264

4. Eke PI, Page RC, Wei L, et al (2012) Update of the Case Definitions for Population-Based Surveillance of Periodontitis. J Periodontol 83:1449–1454. https://doi.org/10.1902/jop.2012.110664

5. Boutaga K, Winkelhoff AJ Van, Vandenbroucke-Grauls CMJE, Savelkoul PHM (2005) Periodontal pathogens: a quantitative comparison of anaerobic culture and real-time PCR. FEMS Immunol Med Microbiol 45:191–199. https://doi.org/10.1016/J.FEMSIM.2005.03.011

6. Altschul SF, Gish W, Miller W, et al (1990) Basic local alignment search tool. J Mol Biol 215:403–410. https://doi.org/10.1016/S0022-2836(05)80360-2

7. Sanger F, Coulson AR (1975) A rapid method for determining sequences in DNA by primed synthesis with DNA polymerase. J Mol Biol 94:. https://doi.org/10.1016/0022-2836(75)90213-2

**Table 1 Supp.** Primers by Beacon designer 8 software and polymorphism variants with their location

| **Gene/ reference SNP** | **Sequence 5’ to 3’** | **Alleles** | **Localization (chromosome)*** |
| --- | --- | --- | --- |
| (DKK) rs1896368 | F: GACACCCAGTCTTAGAAA  R: AGAATCCTTACACGAGTTA | C>T | 10:52309144 a 5266 pb of extreme 3´ |
| (DKK) rs1896367 | F: AGTAATAATCATTGACACTGA  R: TCCAGGCACTTAATACTT | C>T | chromosome 10:52309426, a 4984 of extreme 3´ |
| (DKK) rs1528873 | F: GAGTGGTCAGGAATAAGA  R: GCATTGTTAAGGCTATCA | A>C | chromosome 10:52342245 a 25413 pb of extreme 5´ |
| (SOST475) rs6503475 | F: GTGTAATGGTAAGTATAGC  R: TTTGTAGCCAGTTGA | G>A | chromosome 17:43721456, a 33844 pb near 3´ |
| (KREMEN) rs132274 | F: CTGACAGCAG  R: CTGTATAAATAATCTCTAAAGGTGC | C/T | chromosome 22:29129138 |
| (LRP5) rs3736228 | F: AGGCAGACTGTCAGGACCGCT  R: AGGGTCTTGGCAGAGCCTTGA | C/T | chromosome 11:68433827 |
| *<https://www.ncbi.nlm.nih.gov/clinvar/variation/258640/> | | | |

**Table 2 Supp**. Description of socio-demographic and clinical variables of the study population

| **Variable** | **Healthy-Gingivitis n = 67** | **Periodontitis**  **N = 90** | ***p* value** |
| --- | --- | --- | --- |
| **Age**  Median (IQR) | 34 (25–47) | 47 (36–54) | <0.0001** |
| **Gender** F (%)  Female  Male | 50 (74,6)  17 (25,4) | 66 (73.3)  24 (26.7) | 0.85 |
| **Comorbidity** F (%)  Presence  Absence | 18(26.9)  49(73.1) | 36(40)  54(60) | 0.08 |
| **BMI** F (%)  Normal  Overgrowth  Obesity | 48(71.6)  18(26.9)  1(1.5) | 55(61.1)  27(30)  8(8.9) | 0.11 |
| **Current smoker** F (%)  Current  No | 8(11.9)  59(88.1) | 8(8.9)  82(91,1) | 0.35 |
| **ESR** F (%)  <20  ≥20 | 59(88,1)  8(11,9) | 72(80)  18(20) | 0.32 |
| **CRP** F %  Normal  >3 mg/L | 45 (69,2)  22 (32,8) | 64(71.1)  26 (28.9) | 0.79 |
|  |  |  |  |
| **RF** F %  Normal  **20 U** | 64 (95.5)  3 (4,5) | 81 (90)  9 (10) | 0.42 |

BMI= Body mass index; ESR= Erythrocyte Sedimentation Rate; CRP= High Sensitivity C-Reactive Protein; RF= Rheumatoid factor. IQR= interguartile range; percentille 25 - 75. ***p*< 0.0001

**Table 3 Supp**. DKK rs1896367 genotype association with the severity of periodontal disease

| **Dependent variable** |  | **Unadjusted OR CI 95%** | **Adjusted OR CI 95%** |
| --- | --- | --- | --- |
| **Severe periodontitis** | **Reference** |  |  |
| **Model 1**  **Independent variable** |  |  |  |
| DKK 1896367 TT  DKK 1896367 CT | Negative  Negative | 0.60 (0.17–2.11)  0.24 (0.11–0.53) | 0.76 (0.19–2.95)  0.43 (0.12–1.57) |
| Age > 45 years | < 45 years | 4.45 (2.13–9.26) | 7.69 (2.39–24.7) |
| *P. gingivalis* | Negative | 2.95 (1.40–6.18) | 3.60 (1.15–11.2) |

**Model 1:** Adjusted by age, *P. gingivalis*, smoking history, and BMI. Likelihood ratio= 0.44. BIC unadjusted model= 203.91, BIC adjusted model= 212.38. The unadjusted model must be reported.

**Table 4 Supp**. **Description of frequencies of genetic variants in individuals with periodontal disease**

|  |  | Periodontal disease | |  |
| --- | --- | --- | --- | --- |
|  |  | **Absence** | Presence |  |
|  |  | **N (%)** |  | ***P value*** |
| DKK367 | Absence | 22 (32,8%) | 48 (53.4%) | *0,013^*^* |
|  | Presence | 45 (67,2%) | 42 (46.6%) |  |
| DKK367 | Native | 22 (32,8%) | 48 (53.4%) | *0,039^*^* |
|  | Homozygous* | 6 (9,0%) | 8 (8.9%) |  |
|  | Heterozygous^+^ | 39 (58,2%) | 34 (37.7%) |  |
| DKK368 | Absence | 18 (26,9%) | 13 (14.4%) | *0,040** |
|  | Presence | 49 (73,1%) | 77 (85.6%) |  |
| DKK368 | Native | 18 (26,9%) | 13 (14.5%) | *0,135* |
|  | Homozygous* | 20 (29,9%) | 35 (38.9%) |  |
|  | Heterozygous^+^ | 29 (43,3%) | 42 (46.6%) |  |
| KREMEN | Absence | 12 (17,9%) | 31 (34.4%) | *0,005** |
|  | Presence | 55 (82,1%) | 59 (65.6%) |  |
| KREMEN | Native | 12 (17,9%) | 31 (34.4%) | *0,025** |
|  | Homozygous* | 26 (38,8%) | 22 (24.4%) |  |
|  | Heterozygous^+^ | 29 (43,3%) | 37 (41.2%) |  |

* *p ˂0,05 ** Homozygous gene variant ^+^ Heterozygous gene variant
